# Biogenic hydrogen and methane production from *Chlorella vulgaris *and *Dunaliella tertiolecta *biomass

**DOI:** 10.1186/1754-6834-4-34

**Published:** 2011-09-26

**Authors:** Aino-Maija Lakaniemi, Christopher J Hulatt, David N Thomas, Olli H Tuovinen, Jaakko A Puhakka

**Affiliations:** 1 Department of Chemistry and Bioengineering, Tampere University of Technology, PO Box 541, FI-33101 Tampere, Finland; 2 School of Ocean Sciences, College of Natural Sciences, Bangor University, Menai Bridge, Anglesey LL59 5AB, UK; 3 Finnish Environment Institute, Marine Centre, PO Box 140, FI-00251 Helsinki, Finland; 4 Department of Microbiology, Ohio State University, Columbus, OH 43210, USA

## Abstract

**Background:**

Microalgae are a promising feedstock for biofuel and bioenergy production due to their high photosynthetic efficiencies, high growth rates and no need for external organic carbon supply. In this study, utilization of *Chlorella vulgaris *(a fresh water microalga) and *Dunaliella tertiolecta *(a marine microalga) biomass was tested as a feedstock for anaerobic H_2 _and CH_4 _production.

**Results:**

Anaerobic serum bottle assays were conducted at 37°C with enrichment cultures derived from municipal anaerobic digester sludge. Low levels of H_2 _were produced by anaerobic enrichment cultures, but H_2 _was subsequently consumed even in the presence of 2-bromoethanesulfonic acid, an inhibitor of methanogens. Without inoculation, algal biomass still produced H_2 _due to the activities of satellite bacteria associated with algal cultures. CH_4 _was produced from both types of biomass with anaerobic enrichments. Polymerase chain reaction-denaturing gradient gel electrophoresis profiling indicated the presence of H_2_-producing and H_2_-consuming bacteria in the anaerobic enrichment cultures and the presence of H_2_-producing bacteria among the satellite bacteria in both sources of algal biomass.

**Conclusions:**

H_2 _production by the satellite bacteria was comparable from *D. tertiolecta *(12.6 ml H_2_/g volatile solids (VS)) and from *C. vulgaris *(10.8 ml H_2_/g VS), whereas CH_4 _production was significantly higher from *C. vulgaris *(286 ml/g VS) than from *D. tertiolecta *(24 ml/g VS). The high salinity of the *D. tertiolecta *slurry, prohibitive to methanogens, was the probable reason for lower CH_4 _production.

## Background

Photosynthetic biomass-based fuels are widely considered as viable contenders as sustainable alternatives to fossil fuels. Currently, the major share of biofuels and other forms of bioenergy are produced from terrestrial plants [1]. Microalgae may prove an alternative to terrestrial crops because they have higher photosynthetic efficiencies, higher yields and growth rates, and fewer requirements for cultivation land and they can be grown in saline waters and in arid and barren land areas [1,2]. Microalgal biomass is potent for anaerobic conversion as it can have a high content of lipids, carbohydrates and proteins, and does not contain recalcitrant lignin [1-3]. However, the robust cell walls of some microalgal species may limit digestibility [4,5].

Anaerobic digestion of microalgal biomass for CH_4 _production has been studied at various temperatures and with various pretreatments and cosubstrates [4,6-9]. For example, Chen and Oswald [4] reported that pretreatment of algal biomass at 100°C for 8 h increased digestibility by up to 33%, but the energy consumed in pretreatment was higher than the enhancement gained in CH_4 _production [8].

Some green microalgae, such as *Chlamydomonas reinhardtii *[10] and *Chlorella salina *[11] produce hydrogen under anaerobic conditions via direct photolysis [12]. However, despite extensive research this process has low yields and is rather feeble. It is filled with metabolic and technical obstacles [13] and remains an unlikely source of sustainable energy. Indirect photolysis of microalgal biomass by first hydrolyzing the biomass with lactic acid bacteria followed by photosynthetic H_2 _production resulted in H_2 _yields up to 8 mol H_2_/mol starch glucose from *C. reinhardtii *(66% starch conversion efficiency) [14]. Carver *et al*. [5] reported H_2 _production from dark fermentation of *Chlorella vulgaris *and *Dunaliella tertiolecta *at 60°C. Further, Gfeller and Gibbs [15], Miura *et al*. [16] and Ueno *et al*. [17] reported hydrogen fermentation by microalgal cells under dark, anaerobic conditions.

The aim of this study was to examine the formation of H_2 _and CH_4 _from microalgal biomass. Two green microalgae, *Chlorella vulgaris *(a freshwater species) and *Dunaliella tertiolecta *(a marine species) were used as feedstocks. Experiments were carried out in batch bottles at 37°C without pretreatment of the algal biomass, and the microbial communities were characterized by polymerase chain reaction-denaturing gradient gel electrophoresis (PCR-DGGE) profiling of 16S rRNA gene and sequencing.

## Results

### Algal biomass feedstocks

The chemical composition of the two microalgal biomass feedstocks was different. *C. vulgaris *contained 36%, 13% and 8% of proteins, lipids and sugars on a dry weight basis, respectively. The corresponding mass composition of *D. tertiolecta *was 15%, 11% and 4%, respectively. In general, these values are lower than previously reported in the literature (Additional file 1, Table S1). The compositional data for *D. tertiolecta *in particular may reflect loss of cellular constituents upon sample preparation and handling because the marine microalga does not have a rigid wall and is prone to lyse when the osmotic pressure changes. Growth conditions were not varied to determine the corresponding changes in cellular fractions.

### Enrichment cultures

**Figure 1:**
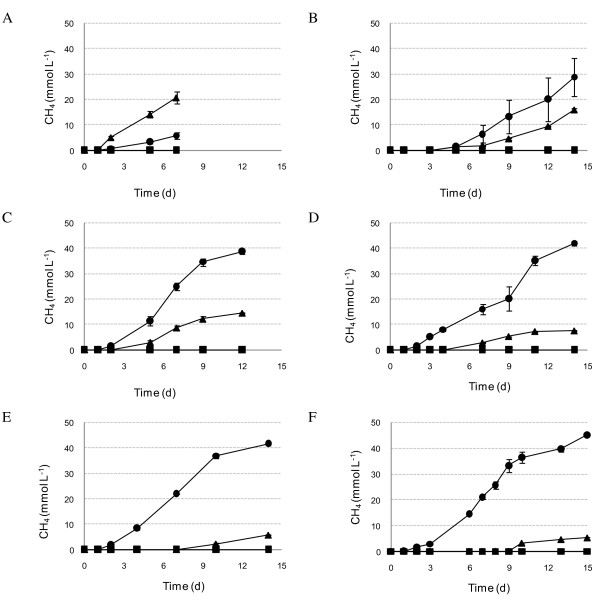
**Methane production during the enrichment**. Methane production (± SE) in enrichment phase 1 **(A)**, 2 **(B)**, 3 **(C)**, 4 **(D)**, 5 **(E) **and 6 **(F) **where closed circles = *Chlorella vulgaris *and U-C, closed squares = *C. vulgaris *and B-C, closed triangles = *Dunaliella tertiolecta *and U-D, and crosses = *D. tertiolecta *and B-D. H_2_-fermenting cultures (with 20 mM BESA): *C. vulgaris *biomass as substrate = B-C; *D. tertiolecta *biomass as substrate = B-D; CH_4_-producing cultures: *C. vulgaris *biomass as substrate = U-C; *D. tertiolecta *biomass as substrate = U-D. Crosses do not show, because they overlap with closed squares.

Four different cultures were enriched from the initial anaerobic digester sludge. Two H_2_-fermenting cultures, one with *C. vulgaris *biomass, designated as B-C, and one with *D. tertiolecta *biomass as the substrate, B-D, and two CH_4_-producing cultures, one utilizing *C. vulgaris *biomass, U-C, and one *D. tertiolecta *biomass, U-D. Methanogenesis was suppressed in the H_2_-fermenting cultures by addition of 20 mM 2-bromoethanesulfonic acid (BESA). During enrichment phases 1-5 no H_2 _was produced in any of the cultures, while in enrichment phases 6 to 9 low levels of H_2 _were detected in B-C and B-D enrichments during the first few days, but usually by day 5 the H_2 _level had decreased below detection limit (results not shown). No CH_4 _was produced in the cultures with added BESA (Figure 1).

With U-C and U-D, the CH_4 _production was higher from *D. tertiolecta *biomass than from *C. vulgaris *biomass in the first enrichment phase when tested with a combination of 25% algal biomass and 75% activated sludge (Figure 1A). From phase 2 onwards, when the proportion of algal biomass in the substrate was increased to 50% or higher, CH_4 _production from *C. vulgaris *surpassed that from *D. tertiolecta *(Figure 1B-F). With 100% *C. vulgaris *and *D. tertiolecta *biomass the rates of CH_4 _production ranged between 3.4-6.5 and 1.2-4.9 ml/day and the lag times between 2.6-5.1 and 5.3-10 days, respectively. The CH_4 _yield and CH_4 _production rate decreased and the lag time increased from *D. tertiolecta *as the enrichment proceeded. The CH_4 _yields from *C. vulgaris *remained more or less constant after enrichment phase 4 (Figure 1).

### H_2 _and CH_4 _production potential

**Figure 2:**
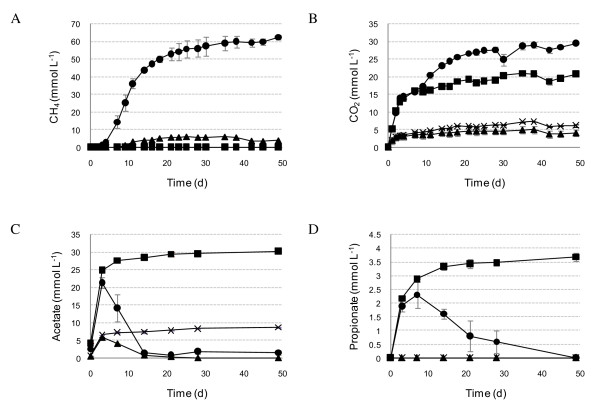
**Metabolite production in cultures with the anaerobic enrichment inocula**. CH_4 _**(A)**, CO_2 _**(B) **and the main fermentation products acetate **(C) **and propionate **(D) **where closed circles = *Chlorella vulgaris *and U-C, closed squares = *C. vulgaris *and B-C, closed triangles = *Dunaliella tertiolecta *and U-D, and crosses = *D. tertiolecta *and B-D. H_2_-fermenting cultures (with 20 mM BESA): *C. vulgaris *biomass as substrate = B-C; *D. tertiolecta *biomass as substrate = B-D; CH_4_-producing cultures: *C. vulgaris *biomass as substrate = U-C; *D. tertiolecta *biomass as substrate = U-D.

**Figure 3:**
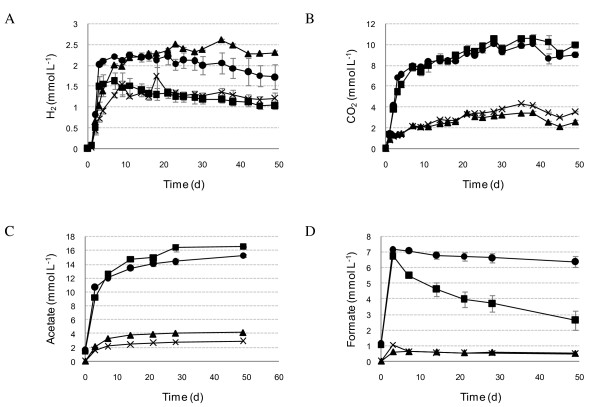
**Metabolite production in the cultures without anaerobic enrichments**. H_2 _**(A)**, CO_2 _**(B) **and the main fermentation products acetate **(C) **and formate **(D) **where closed circles = *Chlorella vulgaris *and no inoculum, closed squares = *C. vulgaris*, 2-bromoethanesulfonic acid (BESA) and no inoculum, closed triangles = *Dunaliella tertiolecta *and no inoculum, and crosses = *D. tertiolecta*, BESA and no inoculum.

Gas production potential from *C. vulgaris *and *D. tertiolecta *was studied using the enrichment cultures after nine passages. Some CO_2 _was produced in all bottles indicating degradation in all cultures, including all controls with no anaerobic inoculum (Table 1, Figures 2B and 3B). CO_2 _production was higher from *C. vulgaris *compared to *D. tertiolecta*.

**Table 1:** Metabolite production in all cultures: cumulative gas production and accumulation of metabolites in the test cultures after 49 day of incubation

Substrate	Inoculum	**H**_**2 **_**(ml)**	**CH**_**4 **_**(ml)**	**CO**_**2 **_**(ml)**	Sum of VFA and alcohols (mM)
None	U-C	0.0 ± 0.0	0.2 ± 0.3	2.3 ± 0.4	-0.6 ± 0.7
None	B-C	0.0 ± 0.0	0.0 ± 0.0	4.7 ± 0.2	5.1 ± 0.5
None	U-D	0.0 ± 0.0	0.0 ± 0.0	1.8 ± 0.0	-0.5 ± 0.1
None	B-D	0.0 ± 0.0	0.0 ± 0.0	4.5 ± 0.2	3.7 ± 0.4
*Chlorella vulgaris*	None	2.1 ± 0.7	0.0 ± 0.0	10.8 ± 0.6	22.1 ± 1.8
*C. vulgaris *and BESA	None	1.3 ± 0.2	0.0 ± 0.0	12.0 ± 0.7	19.1 ± 5.1
*Dunaliella tertiolecta*	None	2.8 ± 0.1	0.0 ± 0.0	3.0 ± 0.4	5.1 ± 0.1
*D. tertiolecta *and BESA	None	1.5 ± 0.3	0.0 ± 0.0	4.2 ± 0.2	4.0 ± 0.3
*C. vulgaris*	U-C	0.0 ± 0.0	74.9 ± 3.6	35.2 ± 0.3	-3.8 ± 1.0
*C. vulgaris*	B-C	0.1 ± 0.0	0.0 ± 0.0	24.8 ± 0.0	31.2 ± 0.7
*D. tertiolecta*	U-D	0.0 ± 0.0	4.7 ± 0.2	4.9 ± 0.0	0.4 ± 0.1
*D. tertiolecta*	B-D	0.0 ± 0.0	0.0 ± 0.0	7.4 ± 0.8	8.9 ± 0.4
Glucose	U-C	4.9 ± 0.4	56.4 ± 0.1	62.5 ± 0.4	2.9 ± 0.7
Glucose	B-C	7.1 ± 0.4	0.0 ± 0.0	57.9 ± 0.9	46.1 ± 0.4
Glucose	U-D	5.2 ± 0.9	38.5 ± 14.0	56.2 ± 7.0	13.3 ± 14.9
Glucose	B-D	14.6 ± 2.3	0.0 ± 0.0	60.8 ± 0.5	44.4 ± 7.1
Cellulose	U-C	0.0 ± 0.0	0.3 ± 0.4	0.9 ± 0.1	0.7 ± 1.4
Cellulose	B-C	0.0 ± 0.0	0.0 ± 0.0	4.0 ± 0.0	7.6 ± 0.4
Cellulose	U-D	0.0 ± 0.0	0.1 ± 0.1	1.8 ± 0.1	0.0 ± 0.1
Cellulose	B-D	0.0 ± 0.0	0.0 ± 0.0	4.6 ± 0.3	5.4 ± 1.3
Chitosan	U-C	0.0 ± 0.0	3.6 ± 4.1	2.4 ± 2.5	0.4 ± 1.5
Chitosan	B-C	0.0 ± 0.0	0.0 ± 0.0	3.1 ± 0.3	6.9 ± 0.5
Chitosan	U-D	0.0 ± 0.0	0.0 ± 0.0	1.0 ± 0.0	0.1 ± 0.1
Chitosan	B-D	0.0 ± 0.0	0.0 ± 0.0	3.2 ± 0.1	5.6 ± 0.8

A minus sign in front of sum of VFA and alcohols indicates that the sum of VFA and alcohols was higher on day 0 than on day 49. The values include standard errors.

H_2_-fermenting cultures: *C. vulgaris *biomass as substrate = B-C; *D. tertiolecta *biomass as substrate = B-D; CH_4_-producing cultures: *C. vulgaris *biomass as substrate = U-C; *D. tertiolecta *biomass as substrate = U-D.

BESA = 2-bromoethanesulfonic acid; VFA = volatile fatty acids.

H_2 _was produced in all cultures including the controls on day 1. With glucose in particular, high levels of H_2 _were produced during first few days. Over time H_2 _decreased to undetectable levels in all cultures except those with algal biomass without inoculum and cultures with glucose and B-D. In the other cultures H_2 _was consumed due to interspecies H_2 _transfer, and cumulative H_2 _production from algal biomass with the anaerobic inocula was negligible (Table 1). With no anaerobic inoculum, H_2 _production was higher from *D. tertiolecta *biomass, 8.4 and 12.6 ml H_2_/g volatile solids (VS), than from *C. vulgaris *biomass, 7.9 and 10.8 ml H_2_/g VS, with and without BESA, respectively. Further enhancement of H_2 _production was attempted by using these cultures as inoculum in batch incubations, but after four enrichment steps no increase in H_2 _production was detected.

No CH_4 _was produced in the cultures amended with BESA (Figure 2). A significant amount of CH_4 _was produced only with *C. vulgaris *and U-C, glucose and U-C, and glucose and U-D (Table 1). Some CH_4 _was also produced with *D. tertiolecta *and U-D as well as with chitosan and U-C (Table 1). CH_4 _production from cellulose was negligible. CH_4 _production from chitosan was significantly lower than that from microalgal biomass. Gas production in controls with no substrate but inoculum was very low, and was taken into account in calculation of the gas production yields (Table 2). Thus, CH_4 _was produced from both *C. vulgaris *and *D. tertiolecta *biomass, while the yield was substantially lower with *D. tertiolecta *than with *C. vulgaris *(Table 2). With *C. vulgaris *biomass 30.6% of organic carbon was released as CH_4 _and 13.6% as CO_2_, while with *D. tertiolecta *biomass the corresponding values were 5.2 and 2.6%, respectively. CH_4 _production from *C. vulgaris *biomass was higher than in glucose controls, while CH_4 _production from *D. tertiolecta *remained far below that of the glucose controls. With glucose, cellulose or chitosan, the H_2 _production was generally higher with the B-D enrichment than with the B-C enrichment, but CH_4 _production was generally higher with the U-C enrichment than with the U-D enrichment (Table 1).

**Table 2:** Production H_2 _and CH_4 _yields from *Chlorella vulgaris *and *Dunaliella tertiolecta *biomass after 49 days of incubation

	**mmol x**^**a **^**per l**	**mmol x**^**a **^**per g volatile solids**	**mmol x**^**a **^**per g added COD**_**tot**_	**mmol x**^**a **^**per g removed COD**_**tot**_
CH_4_				
*C. vulgaris *and U-C	59.6	11.9	5.8	11.3
*C. vulgaris *and B-C	0	0	0	0
*D. tertiolecta *and U-D	5.1	1.0	2.1	3.6
*D. tertiolecta *and B-D	0	0	0	0
H_2_				
*C. vulgaris *and no inoculum	2.3	0.45	0.23	1.7
*C. vulgaris*, BESA and no inoculum	1.6	0.33	0.15	-^b^
*D. tertiolecta *and no inoculum	2.6	0.52	1.6	21.1
*D. tertiolecta*, BESA and no inoculum	1.7	0.35	0.42	4.0

^a^Where x is CH_4 _or H_2_, as indicated.^b^H_2 _yield per g removed COD_tot _could not be calculated as no COD_tot _reduction was detected.

BESA = 2-bromoethanesulfonic acid; COD_tot _= total chemical oxygen demand.

The average chloride ion concentration in the anaerobic incubations was 0.7 and 4.8 g/l and sodium ion concentration was 2.3 and 2.1 g/l in bottles with *C. vulgaris *and *D. tertiolecta *as the substrate, respectively. The pH of the medium was not adjusted at the beginning of the anaerobic incubation. The initial pH was 8.0 in the cultures with algal biomass and 8.5 with the other substrates and the cultures with no substrate. With no substrate, cellulose and chitosan the pH changes were minimal, pH ranging from pH 8.0 to 8.5 during the incubation. With algal biomass, but no inoculum the pH varied between 7.5 and 8.0. With *C. vulgaris *and U-C the pH was 7.5-8.0, with *C. vulgaris *and B-C 7.0-8.0, with *D. tertiolecta *and U-D 8.0-8.5, and with *D. tertiolecta *and B-D 7.5-8.0. In cultures with glucose the pH varied between 6.0 and 8.5.

Organic acids accumulated in the cultures with the B-C and B-D enrichments as well as in the cultures with no anaerobic enrichment inoculum. In the cultures inoculated with U-C and U-D organic acids accumulated only at the beginning of the incubation and were later reduced to CH_4 _(Figure 2C,D). In some cultures, such as with *C. vulgaris *and U-C, the volatile fatty acids (VFA) and ethanol concentrations were lower on day 49 than on day 0 (Table 1). The total concentrations of the soluble degradation products were lower with *D. tertiolecta *than with *C. vulgaris *(Table 1). The main VFA in the anaerobic inocula were acetate and propionate (Figure 2C,D), and acetate and formate in the cultures with no inoculum (Figure 3C,D).

**Figure 4:**
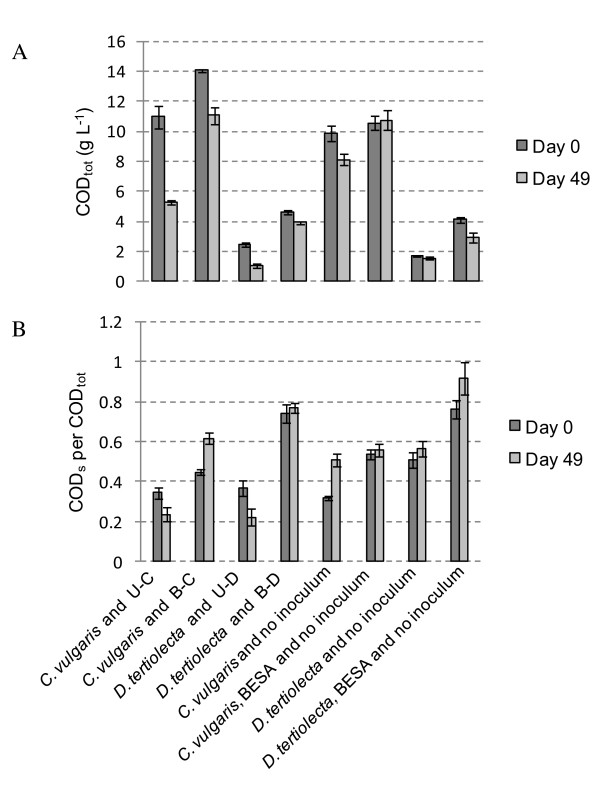
**Chemical oxygen demand (COD) concentrations in cultures with algal biomass**. Total COD (COD_tot_) concentrations and the percentage of soluble COD (COD_s_) at the beginning and end of the cultivation in the cultures with *Chlorella vulgaris *and *Dunaliella tertiolecta *biomass.

The initial total chemical oxygen demand (COD_tot_) values were significantly higher in cultures with *C. vulgaris *than with *D. tertiolecta *in spite of identical initial concentrations of algal VS in all cultures. The addition of 20 mM BESA also increased the initial COD concentration. The COD_tot _concentrations decreased in all cultures between days 0 and 49, except in the case of no substrate and in cultures with *C. vulgaris*, BESA and no inoculum. The decrease in COD_tot _was greater in bottles with U-C and U-D than with B-C and B-D as inoculum, respectively (Figure 4A). COD_tot _reduction was 52% with *C. vulgaris *and U-C, and 57% *D. tertiolecta *and U-D, but only 21% with *C. vulgaris *and B-C, and 15% with *D. tertiolecta *and B-D, respectively. The ratio of soluble COD (COD_s_) to COD_tot _decreased with CH_4 _production, but increased in the other cultures (Figure 4B). The COD results were in line with the VFA and alcohol results.

### Microbial community composition

Based on bacterial PCR-DGGE and sequencing, the initial anaerobic inoculum contained bacteria belonging to phyla Firmicutes, Bacteroidetes, Proteobacteria and Chloroflexi (Additional file 2, Table S2). No genus and species level information for these bacterial sequences were obtained from GenBank.

**Figure 5:**
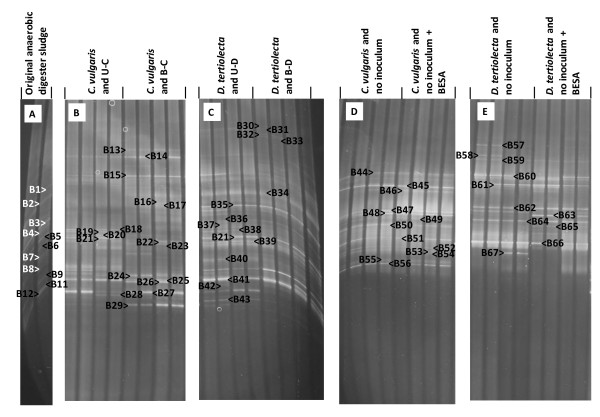
**Polymerase chain reaction-denaturing gradient gel electrophoresis (PCR-DGGE) profiling of the cultures with algal biomass**. Bacterial community profiles from the original anaerobic digester sludge **(A)**, *Chlorella vulgaris *enrichment cultures **(B)**, *Dunaliella tertiolecta *enrichment cultures **(C)**, *C. vulgaris *associated bacteria **(D) **and *D. tertiolecta *associated bacteria **(E)**. See Additional files 2, 3, 4 for the labeled bands.

The bacterial community became enriched during the ten serial batch incubations. Bacterial DGGE profiles were different with the two algal biomass types. The addition of BESA also affected the bacterial community composition (Figure 5). For example, bands B13 and B29 were only clear with *C. vulgaris *and B-C, but not with *C. vulgaris *and U-C. Further, bands B18 and B21 were only clear in *C. vulgaris *and U-C, but not with *C. vulgaris *and B-C. In addition, bands B30-B33 were present in cultures with *D. tertiolecta*, but no corresponding bands were seen in cultures with *C. vulgaris *(Figure 5).

Most of the bacterial 16S rDNA sequences amplified from the anaerobic enrichments matched uncultured bacteria with no species-level information (Additional file 3, Table S3). The matches in the enrichments were *Petrimonas *spp. (band B14), *Bacteroides *spp. (B15), *Bilophila wadsworthia *(B26), *Wolinella succinogenes *(B34), *Oceanibulbus indolifex *(B35), and *Syntrophobacter *spp. (B39). *Petrimonas *spp. were present in all cultures with *C. vulgaris *and anaerobic inoculum, *B. wadsworthia *in *C. vulgaris *and B-C and *Bacteroides *spp. in *C. vulgaris *and B-C as well as in the duplicates of *D. tertiolecta *and U-D. *W. succinogenes*, *O. indolifex *and *Syntrophobacter *spp. were present in all cultures with *D. tertiolecta *and anaerobic inoculum (Figure 4).

A high diversity of bacteria was also present in cultures with no anaerobic inoculum (Figure 4 and Additional File 4, Table S4). These bacteria included *Acidobacterium *spp. (band B44), *Clostridium *spp. (B45, B46, B47, B61), *Clostridium celerecrescens *(B48, B63), *Brevundimonas *spp. (B49), *Hafnia alvei *(B50, B54), *Hafnia alvei *or *Obesumbacterium proteus *(B51), *Gordonia terrae *(B56), *Clostridium sulfidigenes *(B57, B58, B59, B60), *Oceanibulbus indolifex *(B62), *Roseobacter *spp. (B65), *Exiguobacterium *spp. (B66), *Bacillus thermoamylovorans *(B67) and four unknown species (B52, B53, B55, B64).

The DGGE profiles of bacteria associated with *C. vulgaris *and *D. tertiolecta *biomass were different. For example, *H. alvei *was seen only with *C. vulgaris*, whereas *C. sulfidigenes *and B. *thermoamylovorans *only with *D. tertiolecta*. In cultures with *C. vulgaris*, addition of BESA resulted in negligible changes in the bacterial DGGE profile. The only detectable difference was B54 that was identified from the cultures with BESA, but not in the cultures without BESA. In cultures with *D. tertiolecta*, bands B57, B58 and B67 were only visible in cultures without BESA and B63 was significantly brighter with BESA in the medium. Analysis of archaeal 16S rRNA gene sequences was not undertaken in this study.

## Discussion

This work has demonstrated CH_4 _production from *C. vulgaris *and *D. tertiolecta *biomass when inoculated with municipal anaerobic digester sludge enrichments. Biogenic H_2 _was also produced, but it was subsequently consumed without CH_4 _production. H_2 _was produced also in the cultures with algal biomass but no anaerobic inoculum.

H_2 _was produced from both *C. vulgaris *and *D. tertiolecta *biomass by the H_2 _enrichment cultures (containing BESA), but it was subsequently consumed by non-methanogenic microorganisms. The pH was relatively high in these assays. In the cultures with added anaerobic inoculum, H_2 _production was most sustained in the positive controls with glucose, where the pH was also the lowest. Karadag and Puhakka [18] showed with an anaerobic, moderately thermophilic (45°C) enrichment culture that the pH significantly affected H_2 _production from glucose due to pH mediated shifts in fermentation pathways and the bacterial community composition. They reported pH 5.0 was optimal for H_2 _production.

In the present work, several bacteria were identified from the anaerobic inoculum and algal biomass. These included *Petrimonas *spp. that have been previously shown to produce H_2 _[19]. *Syntrophobacter *spp. have been shown to convert propionate to acetate, H_2 _and CO_2_, but only when cocultivated with H_2_-consuming organisms [20,21]. *B. wadsworthia *and *W. succinogenes *utilize H_2 _as their electron donor [22,23]. According to Chassard *et al*. [24]*Bacteroidetes *spp. can suppress H_2 _production from cellulosic material in a mixed culture because they are non-H_2_-producing bacteria with a relatively high cellulolytic activity. *O. indolifex *is an obligately aerobic marine bacterium [25] with no activity under anaerobic conditions and thus it originated from the algal biomass slurry.

H_2 _accumulated in the cultures supplemented only with algal biomass. These cultures formed CO_2 _and accumulated organic acids and alcohols. Gfeller and Gibbs [15], Miura *et al*. [16] and Ueno *et al*. [17] reported hydrogen fermentation by microalgal cells under dark and anaerobic conditions, with H_2 _yields up to 2 mmol H_2_/g dry weight [16]. In this study, H_2 _yields were approximately 25% of that in the cultures with no added anaerobic inoculum (Table 2). However, the DGGE profiles had matches with several H_2_-producing bacteria such as *Clostridium *spp. [26,27] and *Hafnia alvei *[28], which are known H_2 _producers. Some *Bacillus *spp., such as *B. cereus*, *B. thuringiensis *[29] and *B. megaterium *[30] also produce H_2_. According to Combet-Blanc *et al*. [31], *B. thermoamylovorans *does not produce H_2_. *O. proteus *is typical in breweries and is known to cause beer spoilage [32]. Some *Exiguobacterium *spp. such as *E. profundum *are facultatively anaerobic and produce lactate as the main fermentation product [33].

Carver *et al*. [5] used the same algal biomass stocks but a different source inoculum to monitor metabolite production under thermophilic (60°C) conditions. They reported H_2 _production without anaerobic inoculum by heterotrophs associated with *C. vulgaris *biomass, but low H_2 _production with heterotrophs associated with *D. tertiolecta*. In the present study, the *D. tertiolecta*-associated bacteria produced somewhat more H_2_, but approximately 4.5 times less VFA and alcohols and approximately 3 times less CO_2 _than the *C. vulgaris*-associated bacteria. The higher H_2 _production from *D. tertiolecta *was likely due to the lack of proper cell wall in *D. tertiolecta *and differences in bacterial composition of the algal biomass slurry. However, the H_2 _yields reported in this study were low. For comparison, Park *et al*. [34] reported the production of 28 ml H_2 _per g dry weight of the macroalga *Laminaria japonica *pretreated by ball milling and heat treatment at 120°C for 30 min using anaerobic sewage sludge as an inoculum. Carver *et al*. [5] reported production of 82 and 114 ml H_2_/g VS from *C. vulgaris *and 39 and 58 ml H_2_/g VS from *D. tertiolecta *by only microalgal associated bacteria and by a thermophilic consortium at 60°C, respectively.

In the cultures with no added anaerobic inoculum, H_2 _production was somewhat lower with BESA in the medium. This indicates that BESA was inhibitory to some bacteria involved in fermentation. Bacteria present in cultures with no added anaerobic inoculum were associated with the algal culture or were introduced during handling of the biomass.

CH_4 _was produced from both *C. vulgaris *and *D. tertiolecta *biomass, but the yields were not comparable. CH_4 _production was approximately 12 times higher from *C. vulgaris *than from *D. tertiolecta *per added VS but only approximately 3 times higher per added or removed COD_tot _(Table 2). Based on the chemical composition (protein, lipid and sugar content) of the two algal biomass feedstocks, theoretical CH_4 _yield according to Sialve *et al*. [35] would be 463 and 261 ml CH_4_/g VS from *C. vulgaris *and *D. tertiolecta*, respectively. The CH_4 _yields obtained (286 and 24 ml CH_4_/g VS) were 62% and 9% of the theoretical for *C. vulgaris *and *D. tertiolecta*, respectively. However, the cellular composition and major cellular fractions are greatly influenced by storage and culture conditions and cell age. Storage enhances cellular leakage, which was more pronounced with *D. tertiolecta *than with *C. vulgaris*. Based on Becker [36], *C. vulgaris *composition varies on average in the range of 51% to 58% protein, 14% to 22% lipids, and 2% to 17% carbohydrate on dry weight basis. Sydney *et al*. [37] reported 29% proteins, 11% lipids and 14% sugars for *D. tertiolecta *and the closely related *D. salina *contains 57% protein, 6% lipids, and 32% carbohydrate [36]. Additional file 1, Table S1 is a compilation of composition data pooled from specific studies; it is apparent that the bulk cellular composition is a variable parameter.

The large difference in CH_4 _production between the two algal biomasses was likely due to inhibition of digester sludge enrichment by the salinity in the marine *D. tertiolecta *slurry flocculated with NaOH [6,35,38]. Salt toxicity towards methanogens is generally caused by the cation portion of the salt [38], which in this case is Na^+^. For example, McCarty [38] has reported 3.5 to 5.5 g/l Na^+ ^to be moderately toxic and concentrations above 8 g/l highly toxic to methanogens. Similarly, high Cl^- ^levels can also cause inhibition of non-marine methanogens. The levels of dissolved Na^+ ^in cultures with *D. tertiolecta *in this study were 2.1 g/l indicating non-toxic levels of Na^+^. However, Cl^- ^concentration was significantly higher in cultures with *D. tertiolecta *than with *C. vulgaris *as the feedstock. It was also clearly seen from freeze-dried *D. tertiolecta *that salts were bound on the surface of the biomass. Similar salt precipitation was not seen in *C. vulgaris *biomass. Another reason for low CH_4 _production from *D. tertiolecta *biomass may be that *W. succinogenes *was identified from cultures with *D. tertiolecta *and U-D, but not from cultures with *C. vulgaris *and U-C. Coexistence of *W. succinogenes *has been reported to markedly reduce CH_4 _production [39].

Chen and Oswald [4] reported 320 ml CH_4_/g VS from biomass of a mixed microalgal culture from high-rate sewage stabilization ponds heat treated at 100°C for 8 h. Yen and Brune [8] reported 143 ml CH_4_/g VS from an algal mixture including *Scenedesmus *spp. and *Chlorella *spp. without pretreatment. Thus, the CH_4 _yield achieved from *C. vulgaris *was comparable with previous results, but the yield from *D. tertiolecta *was very low. *C. vulgaris *biomass also contained some chitosan, used in flocculation of the biomass. Co-digestion of algal biomass (N-rich material) with C-rich material such as cellulose or chitosan may enhance digestibility [8]. However, the anaerobic enrichments used in this study were not able to utilize chitosan very efficiently. Thus the co-digestion effect was negligible and CH_4 _was mainly produced from the algal biomass.

The calorific yields calculated for the maximum H_2 _and CH_4 _yields were 0.14 kJ/g VS (for H_2 _production from *D. tertiolecta *without added anaerobic inoculum) and 10 kJ/g VS (for CH_4 _production from *C. vulgaris *with enriched digester sludge without BESA). Hydrolytic pretreatment of algal slurries could substantially improve H_2 _production from complex biomass substrate. *C. vulgaris *biomass was shown to be amenable to methanogenic digestion without pretreatment, whilst the high salt content of *D. tertiolecta *biomass likely lowered the CH_4 _yields. However, based on COD_tot_, approximately 50% of *C. vulgaris *biomass was degraded during methanogenic fermentation. Therefore, pretreatment could also enhance CH_4 _production from the biomass of thick cell walled algae, such as *C. vulgaris*, but the energy cost of the pretreatment need to be considered.

## Conclusions

CH_4 _was produced from *C. vulgaris *and *D. tertiolecta *biomass by mesophilic municipal anaerobic digester sludge enrichments. H_2 _was also produced with the anaerobic enrichments but was concurrently consumed by non-methanogenic microorganisms. H_2 _was produced by satellite bacteria associated with algal biomass. PCR-DGGE profiling demonstrated the presence of H_2 _producing (for example, *Petrimonas *spp., *Syntrophobacter *spp.) and H_2 _consuming bacteria (for example, *Bilophila wadsworthia*, *Wolinella succinogenes*) in the anaerobic enrichments and H_2 _producing bacteria (for example, *Clostridium *spp., *Hafnia alvei*) among the satellite bacteria of both microalgal biomasses. H_2 _production by the satellite bacteria was comparable from *D. tertiolecta *and from *C. vulgaris*, but CH_4 _production by the anaerobic enrichments was substantially higher from *C. vulgaris *than from *D. tertiolecta*. The CH_4 _yield obtained from *D. tertiolecta *biomass with the inoculum originating from anaerobic digester was likely limited by the high salinity of the biomass, while the low protein, lipid and carbohydrate content of the *D. tertiolecta *further lowered the CH_4 _yield.

## Methods

### Microalgal biomass production and harvest

*Chlorella vulgaris *(Culture Collection of Algae and Protozoa, UK strain 211/11B) and *Dunaliella tertiolecta *(Sammlung von Algenkulturen Göttingen, Germany, strain SAG 13.86) were grown photoautotrophically in 20 l column (diameter 0.16 m) photobioreactors with 0.5 vvm air sparging and photosynthetically active radiation at photon flux density averaging 225 μmol/m^2^/s. *C. vulgaris *was grown in milliQ-water-based Jaworski's medium (http://www.ccap.ac.uk/media/recipes/JM.htm) and *D. tertiolecta *in natural seawater from the Menai Strait, UK, treated by filtration (0.2 μm) and UV irradiation, with nutrients supplied according to Walne's medium (http://www.ccap.ac.uk/media/documents/Walnes.pdf).

Algal biomass was harvested from 20 l cultures by flocculation followed by centrifugation. *C. vulgaris *was harvested by adding a chitosan stock solution (4 g chitosan, 50 ml acetic acid, 950 ml water) to the culture at approximately 2% of the total volume and adjusting pH to 7 by adding 3 M NaOH to initiate the flocculation. *D. tertiolecta *was flocculated by adding 50-100 ml of 3 M NaOH to raise the pH to approximately pH 9.5 [40]. The biomass of both species was then collected and centrifuged at 1,000 *g *for 10 min to produce a thick paste. The pH of *C. vulgaris *and *D. tertiolecta *biomass was adjusted to 7.0 ± 0.2 with HCl and the biomass slurries were stored at -20°C until used in the gas production experiments. The algal biomass stocks were normalized by measurements of VS.

### Experimental conditions

Anaerobic inocula were enriched from an anaerobic digester treating municipal wastewater sludge (City of Tampere, Finland). Serum bottle enrichments were prepared as series of batch incubations at 37°C with 5 g VS/l of substrate. In the first three phases the substrate consisted of 25% (VS/VS) algal biomass and 75% (VS/VS) of activated sludge, followed by 50% of algal biomass and 50% of activated sludge, and finally 75% of algal biomass and 25% of activated sludge. In the following enrichment phases, 100% of algal biomass was used. Four different cultures were enriched. Two H_2_-fermenting cultures, one with *C. vulgaris *biomass, designated as B-C, and one with *D. tertiolecta *biomass as the substrate, B-D, and two CH_4_-producing cultures, one utilizing *C. vulgaris *biomass, U-C, and one *D. tertiolecta *biomass, U-D. Methanogenesis was suppressed in the H_2_-fermenting cultures by addition of 20 mM BESA. The medium was prepared according to Zehnder *et al*. [41] with modifications by Karlsson *et al*. [42] and Ejlertsson *et al*. [43].

Gas production potential from *C. vulgaris *and *D. tertiolecta *was studied after nine passages of the corresponding enrichment culture at 37°C in 120 ml anaerobic serum bottles with 50 ml of medium and 10% (v/v) inoculum. The incubations included two types of negative controls, with inoculum but no substrate and with 5 g VS/l algal biomass but without anaerobic enrichment inoculum. Three types of positive controls were prepared containing enriched anaerobic inoculum and either 5 g/l glucose, 5 g/l cellulose or 5 g/l chitosan.

### Chemical analyses

The VS concentrations of the biomass samples were measured according to the Finnish Standard SFS 3008 [44]. Carbon and nitrogen were measured with Thermo-Electron Flash EA 1112 after drying the samples at 80°C for 72 hours. The elemental analyzer was calibrated using the standards sulfanilamide, 2,5-bis(5'-tert-butyl-benzoxazolyl)thiophene and l-cystine. dl-methionine was used as a reference material. Mass composition of the two microalgal biomass feedstocks was determined with analytical methods generally used in microalgal studies and at least three replicate samples were included in all analyses. The total lipid content of biomass was measured by extracting the lipids from freeze-dried biomass with chloroform/methanol and determining the lipids gravimetrically [45]. The protein composition of the algal biomass was calculated by multiplying the total elemental nitrogen content by 4.44 [46]. Total carbohydrate concentration of the biomass feedstocks was determined by the phenol sulfuric acid method [47]. Prior to biomass analyzes *D. tertiolecta *biomass was washed with 0.5 M ammonium formate.

Gas production was measured according to Owen *et al*. [48]. The headspace gas composition (H_2_, CH_4 _and CO_2_) was measured using Shimadzu gas chromatograph GC-2014 equipped with Porapak N column (80/100 mesh) and a thermal conductivity detector. The temperatures of the oven, injector and detector were 80, 110 and 110°C, respectively. N_2 _was used as carrier gas at a flow rate of 20 ml/min. The formation of organic acids and alcohol (lactate, formate, acetate, propionate, butyrate and ethanol) was analyzed with a Shimadzu HPLC chromatograph with a Shodex Sugar SH1011 column (Showa Denko K.K., Tokyo, Japan) and a refractive index detector (Shimadzu, Kyoto, Japan). Mobile phase was 5 mM H_2_SO_4 _and flow rate 0.9 ml/min. COD was analyzed before (COD_tot_) and after filtration (COD_s_) through 0.45 μm polyester syringe filter (Macherey-Nagel, Düren, Germany) with dichromate method according to standard SFS 5504 [49]. Concentration of dissolved chloride ions was analyzed with Dionex DX-120 ion chromatograph equipped with AS40 auto sampler and IonPac AS23 (4 × 250 mm) anion exchange column. The mobile phase was Na-carbonate/Na-bicarbonate solution containing 4.5 mM/l Na_2_CO_3 _and 3 mM/l NaHCO_3_. Concentration of dissolved sodium ions was analyzed with inductively coupled plasma emission-mass spectrometry according to industry standard DIN EN ISO 17294.

### Microbial community analyses

Duplicate samples of 1.5 ml were taken from the original digester sludge and from batch bottles at the end of the 49-day incubation and stored at -20°C. Prior to DNA extraction samples were pelleted by centrifugation (10,000 *g*, 5 min) and the supernatant removed. DNA was extracted from the pellets with PowerSoil DNA isolation kit (MO BIO Laboratories, Inc., Carlsbad, CA, USA). The extracted DNA sample was used as a template for the PCR. Partial bacterial 16S rRNA genes of the community DNA were amplified by using primer pair GC-BacV3f [50] and 907r [51] as described by Koskinen *et al*. [26]. DGGE was performed with INGENYphorU2×2-system (Ingeny International BV, GP Goes, The Netherlands) using 8% polyacrylamide gels with denaturing gradient from 30% to 70% (100% denaturing solution contains 7 M of urea and 40% formamide). Gels were run at 60°C in 1 × TAE (40 mM Tris, 20 mM acetic acid, 1 mM ethylenediaminetetra-acetic acid (EDTA), pH 8.3) with 100 V for 22 h and stained with SYBR Gold (Molecular Probes Invitrogen, Eugene, OR, USA). The dominant bands were excised from the gels, eluted in 20 μl of sterile water at 4°C overnight, stored at -20°C and reamplified for sequencing. Sequencing was conducted at Macrogen Inc. (Seoul, Korea). Sequence data were analyzed with BioEdit software and compared with sequences in GenBank.

### Calculations

Cumulative H_2 _and CH_4 _production were calculated according to Logan *et al*. [52]. The data were fitted to a modified Gompertz equation [53] by minimizing the square of the measurements and the estimates subtraction to give lag times and H_2_/CH_4 _production rates. The calorific yields from maximum H_2 _and CH_4 _yields were calculated from the lower heating values, 120 MJ/kg for H_2 _and 50 MJ/kg for CH_4_.

## Competing interests

The authors declare that they have no competing interests.

## Authors' contributions

AML carried out the anaerobic cultivations and all related analyses, microbial community analyses, data interpretation, and drafting and completion of the manuscript. CJH carried out microalgal biomass production and harvesting, and the elemental analysis of the harvested biomass and participated in the drafting of the manuscript. DNT participated in the design of microalgal biomass production and reviewed the manuscript. OHT participated in the design of the study and data interpretation, and thoroughly reviewed the manuscript. JAP conceived the study, participated in data interpretation and thoroughly reviewed the manuscript. All authors read and approved the final manuscript.

## Supplementary Material

Additional file 1 **Mass composition of various microalgae**. Mass composition (dry weight basis) data of microalgae pooled from literature sources. Click here for file

Additional file 2 **Bacterial band identities from the initial sludge**. Matches of selected band identities of PCR-denaturing gradient gel electrophoresis (PCR-DGGE) samples from the initial anaerobic digester sludge. Click here for file

Additional file 3 **Bacterial band identities from the cultures with algal biomass and anaerobic enrichment inocula**. Matches of selected band identities of PCR-denaturing gradient gel electrophoresis (PCR-DGGE) samples from cultures with algal biomass and enriched anaerobic inocula. Click here for file

Additional file 4 **Bacterial band identities from the cultures with algal biomass and no anaerobic enrichments**. Matches of selected band identities of PCR-denaturing gradient gel electrophoresis (PCR-DGGE) samples from the cultures with algal biomass and no anaerobic inoculum. Click here for file
