# Supplementary material for: Biogenic hydrogen and methane production from Chlorella vulgaris and Dunaliella tertiolecta biomass
Source: Biotechnol Biofuels. 2011 Sep 26;4:34. doi: 10.1186/1754-6834-4-34 (PMC3193024; doi:10.1186/1754-6834-4-34)
Supplement: Additional file 1 — Mass composition of various microalgae. Mass composition (dry weight basis) data of microalgae pooled from literature sources. [file 1754-6834-4-34-S1.PDF]

Table S1 Mass composition (dry weight basis) data of microalgae pooled from literature sources.

Species	Protein (%)	Lipid (%)	Carbohydrates / sugars (%)	Sum of proteins, lipids and carbohydrates	Reference
Botryococcus braunii	40	33	2	**75**	[S1]
Chaetoceros muelleri	46	12	4	**62**	[S2]
Chaetoceros sp.	43	19	1	**63**	[S2]
Chaetoceros sp.	37	17	6	**60**	[S3]
Chlorella vulgaris	28	31	8	**67**	[S4]
Chlorella vulgaris	41	10	17	**68**	[S1]
Cryptomonas sp.	47	22	4	**73**	[S3]
Dunaliella tertiolecta	29	11	14	**54**	[S1]
Rhodomonas sp.	29	19	9	**57**	[S3]
Skeletonema costatum	28	14	12	**54**	[S3]
Skeletonema sp.	28	13	4	**45**	[S3]
Tetraselmis sp.	30	13	8	**51**	[S3]

References for supplementary Table S1:

S1. Sydney EB, Sturm W, de Carvalho JC, Thomaz-Soccol V, Larroche C, Pandey A, Soccol CR: **Potential carbon dioxide fixation by industrially important microalgae.** *Bioresour Technol* 2010, 101:5892-5896.

S2. Martínez-Fernández E, Acosta-Salmón H, Southgate P:. **The nutritional value of seven species of tropical microalgae for black-lip pearls oyster (*Pinctada margaritifera*, L.) larvae.** *Aquaculture* 2006, 257:491-503.

S3. Renaud SM, Thinh LV, Parry DL: **The gross chemical composition and fatty acid composition of 18 species of tropical Australian microalgae for possible use in mariculture.** *Aquaculture* 1999, 170:147-159.

S4.Cheng Y-L, Juang YC, Liao G-Y, Ho S-H, Yeh K-L, Chen C-H, Chang J-S, Liu J-C, Lee D-J: **Dispersed ozone flotation of *Chlorella vulgaris*.** *Bioresour Technol* 2010, 101:9092-9096.
