# Supplementary material for: Biogenic hydrogen and methane production from Chlorella vulgaris and Dunaliella tertiolecta biomass
Source: Biotechnol Biofuels. 2011 Sep 26;4:34. doi: 10.1186/1754-6834-4-34 (PMC3193024; doi:10.1186/1754-6834-4-34)
Supplement: Additional file 2 — Bacterial band identities from the initial sludge. Matches of selected band identities of PCR-denaturing gradient gel electrophoresis (PCR-DGGE) samples from the initial anaerobic digester sludge. [file 1754-6834-4-34-S2.PDF]

Table S2 Matches of selected band identities of PCR-DGGE samples from the initial anaerobic digester sludge.

Band label[a]	SL[b]	Sim (%)[c]	Affiliation (acc)[d]	Phylum / Family	Origin of the sample with the closest match
B1	386	91.4	Uncultured Firmicutes bacterium (CU926869)	Firmicutes / unknown	A full-scale mesophilic anaerobic digester
B2	392	100	Uncultured Bacteroidetes bacterium (CU918722)	Bacteroidetes / unknown	A full-scale mesophilic anaerobic digester
B3	464	100	Uncultured Deltaproteobacteria bacterium (CU926802)	Proteobacteria / unknown	A full-scale mesophilic anaerobic digester
B4	393	99.2			
B5	434	99.5	Uncultured bacterium (EU542511)	Unknown / unknown	Dechlorinating microbial community from anoxic estuarine sediment
B6	410	91.0	Uncultured Chloroflexi bacterium (CU918692)	Chloroflexi / unknown	A full-scale mesophilic anaerobic digester
B7	359	79.9			
B8	399	89.7	Uncultured Chloroflexi bacterium (CU918692)	Chloroflexi / unknown	A full-scale mesophilic anaerobic digester
B9	408	85.8	Uncultured bacterium (GQ487786)	Unknown / unknown	Microbial community in a groundwater/ surface water redox transition zone
B10	460	99.6	Uncultured bacterium (DQ088231)	Unknown / unknown	A sulfidogenic bioreactor
B11	447	97.4			
B12	431	98.1	Uncultured Firmicutes bacterium (CU922533)	Firmicutes / unknown	A full-scale mesophilic anaerobic digester

[a]Band label in Figure 5
[b]Sequence length
[c]Similarity (%)
[d]Closest species in GenBank database with an accession number
