# Supplementary material for: Biogenic hydrogen and methane production from Chlorella vulgaris and Dunaliella tertiolecta biomass
Source: Biotechnol Biofuels. 2011 Sep 26;4:34. doi: 10.1186/1754-6834-4-34 (PMC3193024; doi:10.1186/1754-6834-4-34)
Supplement: Additional file 3 — Bacterial band identities from the cultures with algal biomass and anaerobic enrichment inocula. Matches of selected band identities of PCR-denaturing gradient gel electrophoresis (PCR-DGGE) samples from cultures with algal biomass and enriched anaerobic inocula. [file 1754-6834-4-34-S3.PDF]

Table S3 Matches of selected band identities of PCR-DGGE samples from cultures with algal biomass and enriched anaerobic inocula.

Band label[a]	SL[b]	Sim (%)[c]	Affiliation (acc)[d]	Phylum / Family	Origin of the sample with the closest match
B13	351	94.0	Uncultured bacterium (CT574432)	Unknown / unknown	A municipal anaerobic sludge digester
B14	488	97.3	*Petrimonas* sp. (GU583826)	Bacteroidetes / *Porphyromonadaceae*	Chinese luzhou-flavor liquor cellar mud
B15	494	94.3	*Bacteroides* sp. (AY554420)	Bacteroidetes / *Bacteroidaceae*	A landfill leachate bioreactor
B16	340	91.8	Uncultured bacterium (GQ203639)	Unknown / unknown	An anaerobic baffled reactor
B17	363	97.0			
B18	463	97.4			
B19	329	99.4	Thermotogales bacterium (HM003101)	Thermotogae / unknown	An environmental sample from temperate climate
B20	382	91.9			
B21	381	86.9			
B22	452	96.0	Uncultured bacterium (GQ203639)	Unknown / unknown	An anaerobic baffled reactor
B23	458	97.8			
B24	429	96.3	Uncultured bacterium (GQ324637)	Unknown / unknown	A sulfidogenic wastewater biofilm
B25	345	95.9	Uncultured Spirochaetes bacterium (CU922720)	Spirochaetes / unknown	A full-scale mesophilic anaerobic digester
B26	348	95.1	*Bilophila wadsworthia* (AB117562)	Deltaproteobacteria / *Desulfovibrionaceae*	Bile metabolism
B27	483	100	Uncultured bacterium (CT574327)	Unknown / unknown	A municipal anaerobic sludge digester
B28	450	97.8	Uncultured bacterium (AB248641)	Unknown / unknown	A mesophilic anaerobic chemostat fed with butyrate
B29	488	98.0	Uncultured spirochete clone (AY648566)	Spirochaetes / unknown	An anaerobic bioreactor processing sulfate-rich waste streams
B30	406	79.8	Uncultured Bacteroidetes bacterium (AB478930)	Bacteroidetes / unknown	A cassette-electrode microbial fuel cell
B31	450	84.0	Uncultured bacterium (FN563280)	Unknown / unknown	A mesophilic and fuzzy logic controlled 2-phase biogas reactor
B32	458	86.9	Uncultured Bacteroidetes bacterium (CU922564)	Bacteroidetes / unknown	A full-scale mesophilic anaerobic digester
B33	437	96.3			
B34	472	100	*Wolinella succinogenes* (NR_025942)	Proteobacteria / *Helicobacteraceae*	Laboratory culture of *Wolinella succinogenes*
B35	456	100	*Oceanibulbus indolifex* (DQ915614)	Proteobacteria / *Rhodobacteraceae*	Not given
B36	462	84.4	Uncultured bacterium (FJ901102)	Unknown / unknown	Reservoir with a high water cut stage
B37	446	96.0	Alphaproteobacterium (GU061126)	Proteobacteria / unknown	The Yellow Sea
B38	414	73.2	Uncultured Thermotogae bacterium (EU722197)	Thermotogae / unknown	Production water from an Alaskan mesothermic petroleum reservoir
B39	487	91.2	*Syntrophobacter* sp. (EU888828)	Proteobacteria / *Syntrophobacteraceae*	An upflow anaerobic sludge blanket reactor degrading propionate
B40	403	76.4	Uncultured bacterium (EF559198)	Unknown / unknown	A mesophilic anaerobic solid waste digester
B41	489	100	Uncultured bacterium (AY667253)	Unknown / unknown	A dechlorinating community resulting from in situ biostimulation in a trichloroethene-contaminated deep, fractured basalt aquifer
B42	456	100			
B43	448	99.8	Uncultured bacterium (FJ645714)	Unknown / unknown	Microbial population treating anaerobically PCP-contaminated waste streams at low temperature

[a]Band label in Figure 5
[b]Sequence length
[c]Similarity (%)
[d]Closest species in GenBank database with an accession number
