# Supplementary material for: Biogenic hydrogen and methane production from Chlorella vulgaris and Dunaliella tertiolecta biomass
Source: Biotechnol Biofuels. 2011 Sep 26;4:34. doi: 10.1186/1754-6834-4-34 (PMC3193024; doi:10.1186/1754-6834-4-34)
Supplement: Additional file 4 — Bacterial band identities from the cultures with algal biomass and no anaerobic enrichments. Matches of selected band identities of PCR-denaturing gradient gel electrophoresis (PCR-DGGE) samples from the cultures with algal biomass and no anaerobic inoculum. [file 1754-6834-4-34-S4.PDF]

Table S4 Matches of selected band identities of PCR-DGGE samples from the cultures with algal biomass and no anaerobic inoculum.

**Band label**[a]	**SL**[b]	**Sim (%)**[c]	**Affiliation (acc)**[d]	**Class / Family**	**Origin of the sample with the closest match**
B44	418	93.8	Uncultured *Acidobacterium* sp. (AB257652)	Acidobacteria / *Acidobacteriaceae*	Endolithic microorganisms from the pores in exposed dolomite rocks in the Piora Valley
B45	461	100	*Clostridium* sp. (DQ479415)	Firmicutes / *Clostridiaceae*	Bacterial communities involved in sulfur cycle in metalliferous organic soils
B46	337	97.0	*Clostridium* sp. (GU195653)	Firmicutes / *Clostridiaceae*	A bifenthrin degrading bacterium isolated from waste water
B47	474	100	*Clostridium* sp. (FJ384378)	Firmicutes / *Clostridiaceae*	A mesophilic anaerobic digestor
B48	434	99.8	*Clostridium celerecrescens* (FM994938)	Firmicutes / *Clostridiaceae*	A H_2 producing anaerobic sequencing batch reactor
B49	306	88.2	*Brevundimonas* sp. (HM777012)	Proteobacteria/ *Caulobacteraceae*	Not given
B50	433	100	*Hafnia alvei* (AB244475)	Proteobacteria/ *Enterobacteriaceae*	Crop of the antlion species *Myrmeleon bore*
B51	388	100	*Hafnia alvei* (AB519795) / *Obesumbacterium proteus* (FJ492810)	Proteobacteria/ *Enterobacteriaceae*	Not given / bacteria associated with brewery yeasts
B52	405	90.1	Uncultured bacterium (EF154421)	Unknown / unknown	Digestive tracts of ground beetles
B53	421	90.0	Uncultured beta proteobacterium (FJ975852)	Unknown / unknown	Human gut microbiome
B54	415	96.6	*Hafnia alvei* (AB519795)	Proteobacteria/ *Enterobacteriaceae*	Not given
B55	421	94.5	Uncultured bacterium (EU803296)	Unknown / unknown	Ocean sample
B56	467	96.6	*Gordonia terrae* (AY771337)	Actinobacteria / *Gordoniaceae*	Laboratory culture collection
B57	474	100	*Clostridium sulfidigenes* (HM163536)	Firmicutes / *Clostridiaceae*	Bacterial communities associated with photosynthetic plants
B58	475	100			
B59	422	97.9			
B60	479	99.8			
B61	488	99.8	*Clostridium* sp. (DQ168187)	Firmicutes / *Clostridiaceae*	Soil from the Florida Everglades
B62	456	100	*Oceanibulbus indolifex* (DQ915614)	Proteobacteria / Rhodobacteraceae	Not given
B63	460	100	*Clostridium celerecrescens* (FM994938)	Firmicutes / *Clostridiaceae*	A H_2 producing anaerobic sequencing batch reactor
B64	363	97.8	Uncultured bacterium (FJ203216)	Unknown / unknown	Bacteria associated with the coral *Montastraea faveolata*
B65	445	99.6	*Roseobacter* sp. (EF512125)	Proteobacteria / *Rhodobacteraceae*	Symbiotic microorganisms in *Isochrysis galbana* culture
B66	461	99.6	*Exiguobacterium* sp. (FN435981)	Firmicutes / *Bacillaceae*	Microbial community inhabiting deteriorated stones
B67	454	97.8	*Bacillus thermoamylovorans* (AB360808)	Firmicutes / *Bacillaceae*	Not given

[a]Band label in Figure 5
[b]Sequence length
[c]Similarity (%)
[d]Closest species in GenBank database with an accession number
